# Social support, network, and relationships among coaches in different sports: a systematic review

**DOI:** 10.3389/fpsyg.2024.1301978

**Published:** 2024-09-24

**Authors:** João Gonçalo Ferreira, Filipe Rodrigues, Pedro Sobreiro, Mário Silva, Fernando Jorge Santos, Gonçalo Carvalho, António Hernández Mendo, José Rodrigues

**Affiliations:** ^1^Sport Sciences School of Rio Maior, Polytechnic University of Santarém, Rio Maior, Portugal; ^2^Life Quality Research Centre, Santarém, Portugal; ^3^ESECS – Polytechnic of Leiria, Leiria, Portugal; ^4^Research Center in Sports, Health, and Human Development, Vila Real, Portugal; ^5^School of Health Sciences, Polytechnic University of Santarém, Rio Maior, Portugal; ^6^School of Education, Polytechnic University of Setúbal, Setúbal, Portugal; ^7^Faculty of Human Kinetics, University of Lisbon, Cruz Quebrada, Portugal; ^8^Department of Social Psychology, Social Anthropology, Social Work and Social Services, University of Malaga, Málaga, Spain

**Keywords:** social support, social network, social relationships, sport, coaching

## Abstract

**Introduction:**

The study aims to analyze scientific publications on the association between social networks, social relationships, and social support for sports coaches. It seeks to identify the types and levels of social support provided by various agents, and to understand the impact of this support on coaches’ wellbeing. The goal is to help coaches better utilize social support, thereby enhancing their quality of life, work, and performance.

**Methods:**

This study systematically reviewed 11 scientific articles to investigate the association between social support, social networks, and social relationships in sports coaches. It aimed to identify the types and levels of social support offered to coaches by family members, peers, and friends. Our research utilized the PRISMA guidelines for systematic reviews and assessed study quality using the STROBE Statement. Eligibility was determined by the PECOS criterion based on the search strategy terms.

**Results:**

Our findings indicate that social support has significant positive effects on sports coaches. It enhances selfcompassion, prevents burnout symptoms, boosts job and life satisfaction, and reduces stress levels. Organizational support, characterized by clear guidelines, guidance, and autonomy, yielded positive outcomes. Conversely, the absence of social support correlated with negative outcomes for coaches, including lower self-compassion, increased stress and burnout symptoms, reduced job and life satisfaction, and heightened work–family conflict. Coaches’ social networks encompassed family members, peers, friends, and other sources, with friends perceived as the most influential. Maintaining an effective social support network is crucial for coaches’ performance and psychological wellbeing.

**Discussion:**

This systematic review emphasizes the importance of social support for coaches in both their personal and professional lives, noting its positive effects and the negative consequences of its absence. Given the demanding nature of coaching, improving social support systems can enhance coaches’ wellbeing and the success of sports activities.

## 1 Introduction

The role of a sports coach is pivotal in enhancing various facets of the athletes, including physical, technical, mental, and tactical aspects, while concurrently playing a crucial role in their personal and social development (Fletcher and Scott, 2010). Given the competitive, intricate, and ever-evolving environment within which sports coaches operate, coupled with the multifaceted responsibilities assigned to them, there has been a burgeoning interest in assessing social support within sports training (Knights and Ruddock-Hudson, 2016; Mannino et al., 2019).

Sports coaching is widely recognized as a demanding profession, characterized by stressors such as long and irregular working hours, job insecurity tied to athletic performance, work–family conflicts, and a significant emotional investment in the coaching role. The accumulation of stress can lead some coaches to experience negative mental health outcomes like burnout, potentially prompting them to leave their positions. However, the impact on mental health varies among coaches, influenced in part by both risk and protective factors. Previous research has predominantly focused on stressors rather than protective factors. Noteworthy protective elements encompass grit, psychosocial resilience, coping skills, self-compassion, and social support. Recent research has shown increasing interest in the latter two factors, as they are believed to enhance overall wellbeing and coping mechanisms in coaches (Ackeret et al., 2022).

The documented benefits of social support in various professions stand in stark contrast to the relatively limited research related to social support among sports coaches (Norris et al., 2020). The existing literature characterizes “social support” as encompassing “comfort, care, assistance, and information that a person receives from others.” In the sports context, social support has been employed to describe the overall quality of relationships, perceived support, availability, or received support, or the size of an individual’s social network (Rees and Hardy, 2000; Bruner et al., 2021). Hobfoll et al. (1990) have underscored the pivotal role of social support and connections within a social network in shielding recipients and preserving their sense of identity.

Like resilient coping, social support is recognized as a protective factor against the adverse effects of negative life experiences. It plays a role in reducing men’s resilient coping for psychological distress by influencing the appraisal of stressful events, fostering a sense of understanding, enhancing control or mastery, and promoting the adoption of adaptive coping strategies. Conversely, a lack of social support can diminish resilience to psychological distress, leading to increased isolation, distancing from social connections, and ineffective self-management (Sharp et al., 2023).

According to Sheikh et al. (2016), social support has an influence on an individual’s wellbeing. Mannino and Caronia (2017) state that wellbeing, often equated with “being well” or “existing well,” encompasses all aspects of human life and defines the quality of life for each individual. It is the result of achieving harmony between people and their environment through adaptation to various lifestyle factors. While it’s often likened to happiness, wellbeing has a broader meaning. It involves an ongoing interaction and mutual influence between individual and collective wellbeing, ultimately leading to individual happiness within the social context.

Social support is a multifaceted concept encompassing emotional, informational, esteem, and tangible dimensions. Emotional support involves providing comfort and security during high-stress times, fostering feelings of love and protection. Informational support includes offering guidance or advice to resolve specific issues, addressing concerns like low confidence or fitness. Esteem support reinforces a person’s competence and self-esteem, aiding in coping with pressure, such as in sports competitions. Tangible support involves concrete instrumental assistance during stressful situations, such as financial support or help with tasks (Rees and Hardy, 2000; Maciel et al., 2021).

On the player’s side, several studies suggest that promoting various forms of social support within the sports context can bolster athletes’ motivation (DeFreese and Smith, 2013), self-confidence (Cowan et al., 2012), perceptions of team cohesion (Westre and Weiss, 1991), and performance (Rees et al., 1999). DeFreese and Smith (2013) and other scholars have proposed that received support can serve to reduce athlete burnout, alleviate perceived stress (Rees and Freeman, 2007), and expedite the recovery process from injuries (Abgarov et al., 2012; Clement and Shannon, 2011; Lu and Hsu, 2013).

Although coaching is acknowledged as a notably stressful, intricate, and challenging profession, the academic focus on social support for coaches has not matched that given to athletes. Coaches at various performance levels are tasked with creating effective training programs, recruiting athletes, handling performance-related stress (e.g., competition outcomes) and managing relationships with diverse stakeholders (e.g., athletes, administrators, officials, media, and parents) on a daily basis. It is evident that social support can serve as a valuable resource for coaches, especially when dealing with stressors, as these stressors can have notable consequences on the psychological wellbeing of coaches (e.g., depression) and performance outcomes (e.g., diminished concentration leading to less effective observations) (Norris et al., 2020).

Social support is frequently characterized by considering both the configuration of an individual’s network (such as the presence of family ties) and the explicit resources that can be offered by interpersonal relationships (Norris et al., 2020). According to Thoits (1995), the structure of social networks encompasses the number of relationships an individual maintains within their network, the frequency of contact with network members, and the density of relationships they maintain within the network.

An individual’s social network comprises primarily family, friends, and peers (Norris et al., 2020). The structure of an individual’s social network, such as the presence of familial bonds, and the potential resources offered by interpersonal relationships are concepts intimately intertwined with the concept of social support (Cohen and Wills, 1985). Wellard (2013) research underscores the significance of social relationships in shaping the sporting experiences of participants, influencing their capacity to derive enjoyment from sports engagement. Social relationships also exert a substantial impact on health, lifestyle (Eime et al., 2013; Marques et al., 2016), as well as fostering feelings of belonging and integration (Burrmann et al., 2017; Doidge et al., 2020; Flensner et al., 2021).

Athletes often express that meeting people and socializing with friends serve as compelling motivations for their participation in sports. Most individuals harbors high expectations regarding the social outcomes of their involvement in sports (Bergesen Dalen and Seippel, 2019). Social relationships have a robust association with physical health (House et al., 1988).

Norris et al. (2017), on a systematic review focusing on stressors, coping mechanisms, and wellbeing among coaches, social support emerged in more than 50% of the 38 studies included in the final sample. The results of this review indicate that coaches commonly rely on social support, and in instances where such support is lacking, coaches tend to report heightened perceptions of stress and diminished performance.

In a meta-analysis, Holt-Lunstad et al. (2010) unearthed a connection between social support and reduced mortality risk, even when accounting for conventional risk factors like age, obesity, and health status. Furthermore, studies by De Vogli et al. (2007) and Friedman et al. (1995) provide evidence that negative social connections are linked to higher mortality rates.

The belief prevails that relationships among individuals are intertwined with sleep quality. Throughout the evolutionary history of our species, sleeping humans have benefited from a secure context, protected from predators and adversaries by those in proximity (Dahl and El-Sheikh, 2007). Studies conducted by Nordin et al. (2008), Rambod et al. (2012), and Troxel et al. (2010) have substantiated the notion that perceived support from others correlates with improvements in both objective and subjective sleep quality. Troxel et al. (2010) study further revealed that individuals perceiving greater social support experienced fewer wakeful periods after falling asleep. Moreover, negativity and interpersonal tension have been associated with sleep quality. A study conducted by Brummett et al. (2006) found that negative emotions linked to caregiving predicted poorer sleep quality, affecting facets such as sleep duration, latency, disturbances, and daytime functionality.

Holt-Lunstad et al. (2010) have provided evidence suggesting that the quantity and quality of social relationships in industrialized societies are on the decline. McPherson et al. (2006) argued that in the United States, the number of people with whom individuals engage in significant conversations has markedly dwindled in recent years, leading to heightened social isolation.

The primary objective of this study is to conduct a comprehensive analysis of scientific publications concerning the interplay between social networks, social relationships, and social support in the context of sports coaches. The aim is to identify the types and levels of social support provided to sports coaches by family members, peers, and friends, understanding the impact that social support will have on various aspects of the coaches’ lives, such as wellbeing. In this way, we intend for coaches to have a greater capacity to explore and benefit from social support, improving their quality of life/work and, consequently, their performance.

## 2 Methods

### 2.1 Research

For this systematic review, the criteria recommended by the Preferred Reporting Items for Systematic Reviews and Meta-Analyses statement were followed, which provide guidance for conducting systematic reviews (Moher et al., 2010, 2015). The research protocol was registered with the International Prospective Register of Systematic Reviews (PROSPERO - CRD42023423875), available at: https://www.crd.york.ac.uk/prospero/display_record.php?ID=CRD42023423875.

### 2.2 Strategy

The literature search was conducted in the following databases: Web of Science - Core Collection; and B-On (Academic Search Complete, Business Source Complete, Complementary Index, eBook Index, ERIC, MEDLINE, OpenDissertations, Supplemental Index, and Teacher Reference Center). In the search engines of each platform, the following terms related to the study’s topic (“Social Support” OR “Social Network” OR “Social Relationships” OR “Peer Support” OR “Family Support”) were entered and combined with terms related to the population (“Coach” OR “Instructor” OR “Monitor”) and the research context “Sport.” Using the Boolean operator “AND” the terms were combined as follows: (“Social Support” OR “Social Network” OR “Social Relationships” OR “Peer Support” OR “Family Support” AND “Coach” OR “Instructor” OR “Monitor” AND “Sport”).

### 2.3 Eligibility criteria

**Figure 1:**
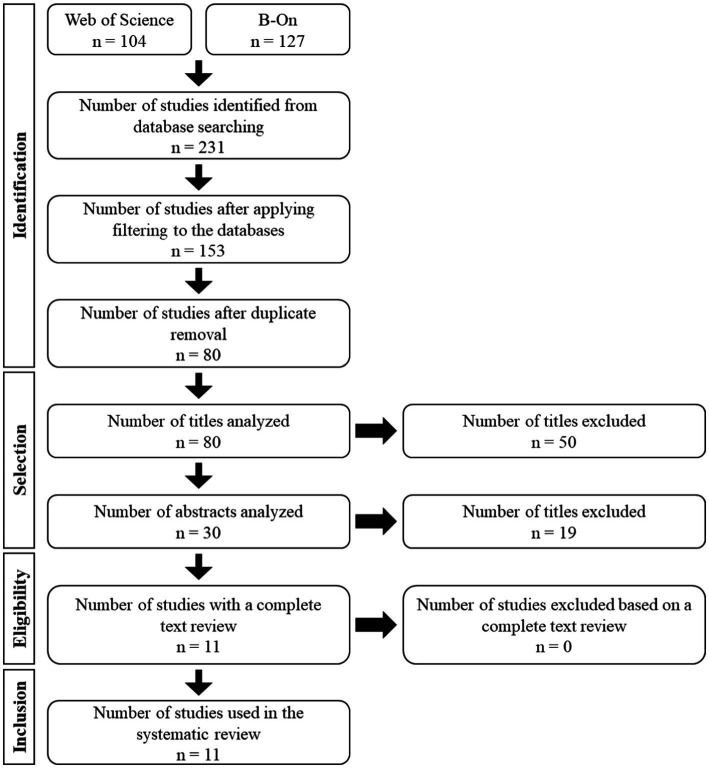
PRISMA flowchart.

The eligibility criteria for the consulted articles followed the recommendations from the literature for such studies (Meline, 2006). For the analysis, all studies in Portuguese, Spanish, and English published between the years 2000 and 2023 (21st century) were considered. Initially, eligibility assessment was conducted by two researchers in the field of sports coaching, in a standardized and independent manner. Subsequently, the assessment by the two researchers was shared with a group of six researchers who performed a new evaluation to ensure consensus among all. The inclusion and exclusion of studies were done using the PECOS criteria (Table 1). In the PECOS criteria, researchers began by excluding studies that involved athletes as participants and included studies that involved coaches and their family, peers, and friends. In the exposure, studies that integrated sports coaching and competition were included, while those related to school physical education were excluded. In the comparison, studies with social agents such as coaches, family, peers, and friends were included. Regarding results, studies that encompassed social support, social networks, social relationships (for/from coaches) (interaction, influence, and association) were included, and consequently, studies addressing the perception of athletes were excluded. Finally, regarding the study itself, those that conducted empirical and instrumental research, both qualitative and quantitative (field studies – descriptive, cross-sectional, and longitudinal), were included, while experimental and theoretical studies were excluded. Figure 1 shows the procedures carried out from the identification stage to the selection of final studies. After searching the selected databases, on February 10, 2023, 231 studies were identified. In the identification phase, filtering was applied to the databases (78 studies excluded due to publication date and document type), and duplicate studies were removed (73 studies excluded). In the screening phase, titles (50 excluded) and abstracts (19 excluded) were read. In the eligibility phase, the remaining studies were read (0 excluded), resulting in 11 studies included in the final stage. Figure 1 depicts the flowchart of the article selection process carried out in this systematic review.

**Table 1:** Inclusion and exclusion criteria for selected studies in the review.

		Inclusion	Exclusion
P	Participant	Coaches (family, peers, and friends)	Athletes
E	Exposure	Sports training and competition	School physical education
C	Comparison	Social agents: family, peers, and friends support type: informational, emotional, tangible, and esteem Support Level: positive, indifferent, and negative	--
O	Outcome	Social support; social network; social relationships (to coaches) (interaction; influence; association)	Athletes’ perception
S	Study	Empirical and Instrumental Investigation (field studies - descriptive, cross sectional and longitudinal)	Experimental and theoretical

### 2.4 Assessment of methodological quality of studies

The methodological quality of each study under analysis was independently assessed by two researchers using the STROBE Checklist (Von Elm et al., 2007). All items from the STROBE Checklist were used, except item 6(b), due to the absence of paired studies. The studies were classified based on the following cut-off points: A (>80% high); B (50–80% moderate); and C (<50% low). The cut-off points were derived from the sum of the scores assigned to each item: 0 (does not address); 1 (addresses) (Olmos et al., 2008). Disagreements between researchers were resolved through consensus. Table 2 presents the results of the methodological quality for the studies included.

**Table 2:** Study characteristics.

Authors and year	Location	Sex	Modality	Instruments	Study quality
Ackeret et al. (2022)	Switzerland	M + F	Individual + Team	Questionnaire	A
Allen and Shaw (2009)	New Zealand	F	Individual + Team	Semi-structured Interview	C
del Prado et al. (2012)	Spain	M + F	Individual + Team	Questionnaire	C
Ha et al. (2021)	South Korea	M + F	Individual + Team	Questionnaire	B
Knights and Ruddock-Hudson (2016)	Australia	M	Team	Semi-structured Interview	C
Teck Koh et al. (2019)	Singapore	M + F	Individual + Team	Semi-structured Interview	B
Kubayi (2018)	South Africa	M + F	Team	Questionnaire	B
Norris et al. (2020)	United Kingdom	M + F	Individual + Team	Semi-structured Interview and Sociograms Assisted by Interviewee	B
Norris et al. (2022)	United Kingdom	M + F	Individual + Team	Semi-structured Interview	B
Occhino et al. (2013)	Australia	M	Team	Semi-structured Interview	C
Pelser-Carstens et al. (2015)	South Africa	M + F	N/A	Questionnaires	B

M, male; F, female; A, high; B, moderate; C, low.

### 2.5 Risk of bias

Based on the guidelines used (Von Elm et al., 2007; Olmos et al., 2008), one study was classified as high methodological quality, six studies were classified as moderate quality, and four studies were classified as low quality. Overall, the risk of bias in the studies was rated as moderate.

### 2.6 Data extraction and analysis

The extracted studies were organized using Zotero software. Based on the information presented in each study, characteristics (publication year, sample type, type of sport, investigated social agents, research type, and instruments) were analyzed.

### 2.7 Study characteristics

The characteristics of the 11 studies selected for this systematic review are presented in Table 2. Of the total number of studies, 8 were published from the year 2015 onward. Regarding the study location, 1 study was conducted in Switzerland, 1 study in New Zealand, 1 study in Spain, 1 study in South Korea, 1 study in Singapore, 2 studies in South Africa, 2 studies in Australia, and 2 studies in the United Kingdom. Most of the studies (*n* = 8) were conducted with participants of both sexes, while 2 studies were conducted only with male participants and 1 study with female participants. Regarding the type of sports, 7 studies included both individual and team sports, 3 studies included only team sports, and 1 study did not specify the types of sports included in the sample. Regarding the instruments used, 5 studies used questionnaires, and 6 studies used semi-structured interviews, with 1 of those studies also using sociograms provided by the interviewee.

## 3 Results and discussion

The primary objective of this systematic review was to explore and analyze the existing literature on social support, social network, and social relationships of coaches across various sports disciplines. To achieve this, we conducted a comprehensive analysis of studies published between 2000 and 2023, focusing on the types of social support received by coaches, the impact of social networks on their wellbeing, and the dynamics of their social relationships with family, peers, and friends.

**Figure 2:**
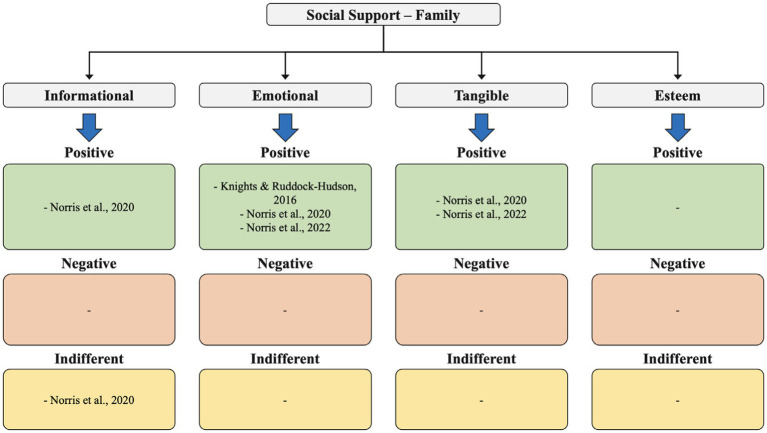
Social support – family.

**Figure 3 fig3:**
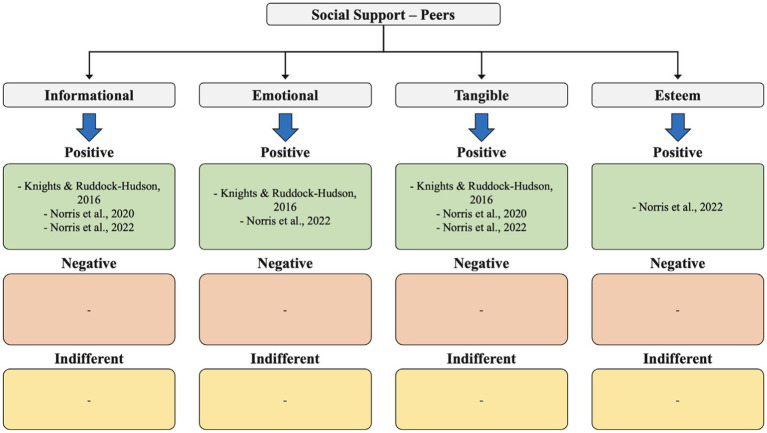
Social support – peers.

**Figure 4:**
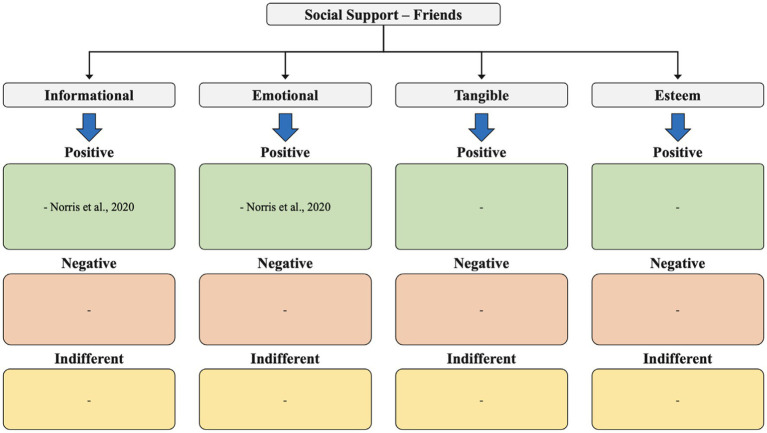
Social support – friends.

In our analysis of the 11 identified studies, we qualitatively assessed the results related to three main themes: social support, social networks, and social relationships. This included examining several types of social support coaches receive (from family, peers, and friends), such as emotional, informational, esteem, and tangible support, as well as the various levels of support (positive, neutral, and negative) perceived by coaches (Figures 2 [Fig fig3]–4).

In the studies analyzed, the social support provided by the family was mainly emotional (3 studies), demonstrating positive effects on the coach’s wellbeing. Tangible support (2 studies) was also positively received by coaches, in situations where they had little time to carry out tasks and resorted to help from family members.

Social support provided by peers was mainly informational (3 studies) and tangible (3 studies), both with positive effects on sports coaches. Informational support was mostly used by coaches to discuss about training sessions with other coaches, while tangible support was based on help from assistant coaches in carrying out tasks.

Friends also had a positive impact on coaches’ wellbeing, providing both informational (1 study) and emotional (1 study) support. Usually, coaches spend time with their friends to “get away” from their working routine, looking to take their mind off work for a while.

**Figure 5:**
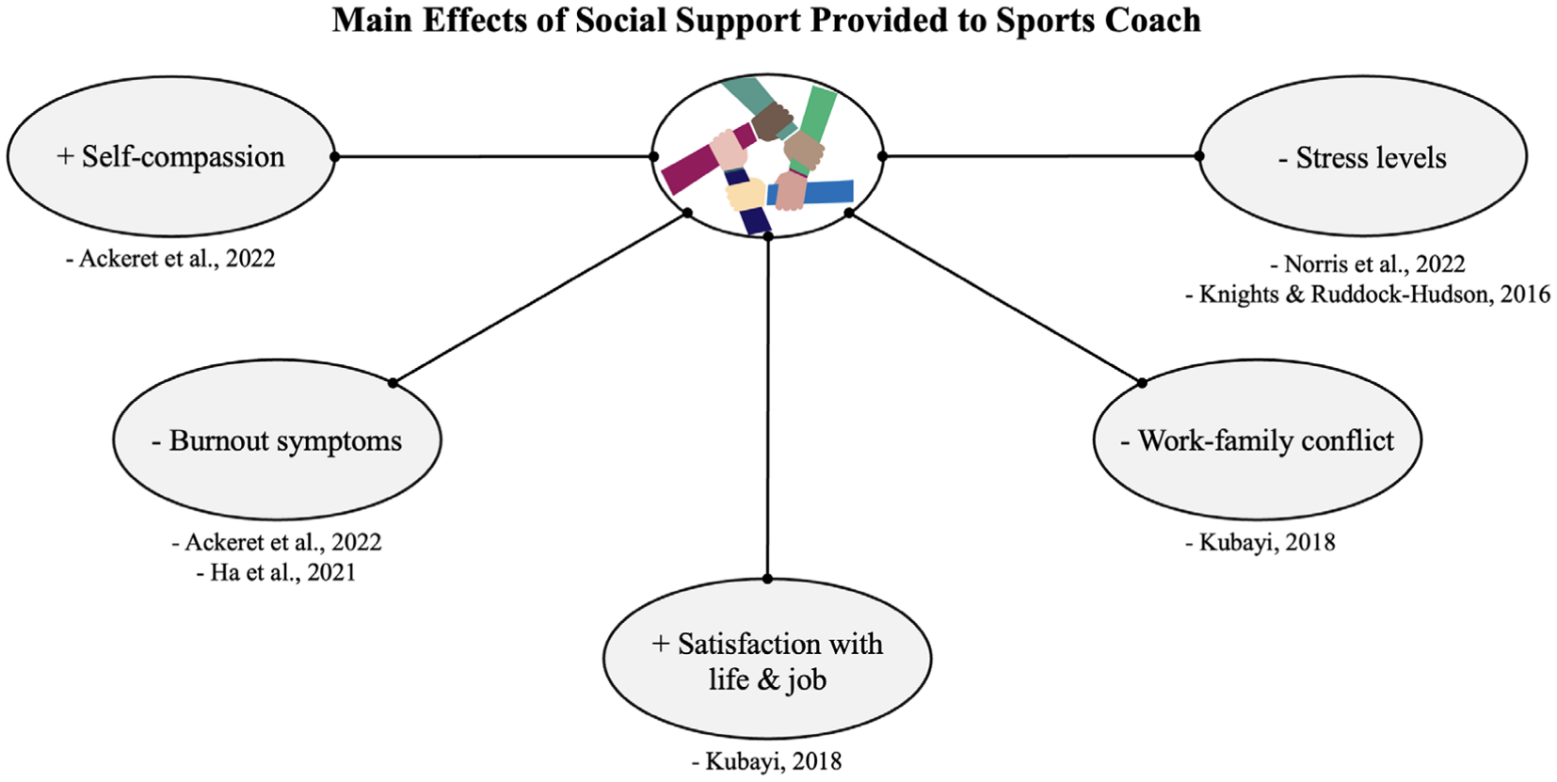
Main effects of social support provided to sports coach.

Within the scope of the studies reviewed, social support emerged as a crucial factor with positive effects on sports coaches across multiple dimensions (Figure 5). Notably, social support was associated with increased self-compassion (Ackeret et al., 2022), the prevention of burnout symptoms (Ackeret et al., 2022; Ha et al., 2021), heightened job and life satisfaction (Kubayi, 2018), and reduced stress levels (Norris et al., 2022; Knights and Ruddock-Hudson, 2016). Additionally, regarding the family, organizational support, was found to have positive effects by reducing work–family conflicts (Kubayi, 2018).

The support provided to coaches by the respective organizations demonstrated positive effects (e.g., coaches felt support for their psychological needs) when there were clear guidelines and guidance, as well as the possibility for coaches to work in contexts of high autonomy. On the other hand, organizations lacking such directives did not reveal such positive results. The extent to which the environment was perceived as autonomy supportive seemed to be significantly influenced by the relationships among coaches and key personnel within sports organizations (Allen and Shaw, 2009).

Findings from studies by Norris et al. (2020), Norris et al. (2022), underscored the importance of all four types of social support (emotional, informational, esteem, and tangible) in yielding positive effects, including stress reduction and improved wellbeing, for coaches. Conversely, the absence of social support tended to result in various negative outcomes in coaches’ lives, such as decreased self-compassion, increased stress and burnout symptoms, job and life dissatisfaction, and heightened work–family conflicts (Ackeret et al., 2022; Ha et al., 2021; Knights and Ruddock-Hudson, 2016; Kubayi, 2018; Norris et al., 2022).

In terms of social networks, studies by Norris et al. (2020) illuminated the comprehensive nature of coaches’ social networks, encompassing colleagues, friends, family members, and other entities (e.g., media). Ego-network diagrams from the study revealed coaches’ tendencies to seek more support from peers, but they often perceived support from friends as the most influential. These diagrams have been created to illustrate the social support utilized by coaches and the proximity of that support. They were formulated based on the sociograms generated from coaches’ interviews. In addition to the ego-network diagrams, insights from interviewees are incorporated through relevant quotes. The results underscored the potential positive impact of maintaining an effective social support network on coaches’ performance and psychological wellbeing.

This systematic review highlighted the significance of social support, social networks, and social relationships in the lives of sports coaches. The findings emphasize the positive effects of social support and the importance of clear guidelines and autonomy within organizations. However, there is a notable gap in scientific literature on this topic, calling for further research to explore and develop a comprehensive conceptual framework integrating social variables and blending quantitative and qualitative methodologies. The study underscores the need for coaches and sports organizations to prioritize social support, considering the competitive and challenging environments coaches navigate, which can otherwise lead to negative consequences for coaches’ wellbeing.

## 4 Conclusion

The results of this systematic review provide valuable insights into the diverse types and levels of social support provided to coaches by individuals in their social networks. It was found that coaches’ social networks encompass colleagues, friends, family, and others. The social support received by coaches was crucial in promoting various positive effects, such as reducing stress, preventing burnout symptoms, increasing job and life satisfaction, and improving work-family relationships. The social support provided by coaches to athletes is also important for maintaining the wellbeing of the athletes. Therefore, it is essential for coaches and sports organizations to invest in providing social support to coaches, given the competitive and challenging environment they operate in, which can lead to negative situations affecting the wellbeing of sports coaches. The study conducted highlights the limited scientific production on the social support of coaches, making it crucial to increase research in this area. The methodologies used stem from research paradigms in psychology, necessitating the development of a new conceptual framework that integrates social variables and blends quantitative and qualitative research methods.

The reviewed studies emphasize the need for coaches and sports club organizational structures to pay attention to this dimension of coaches’ social support. It is the professional responsibility of coaches to ensure support and guidance in their profession. The special attention of family members, athletes, managers, and others to the actions of the coach is crucial for the success of the sports project at hand.

In conclusion, this systematic review underscores the significance of social support for coaches in various aspects of their personal and professional lives. It highlights the positive outcomes associated with social support and the potential drawbacks of its absence. As coaching is a demanding profession, understanding and enhancing social support systems can contribute to the wellbeing of coaches and, subsequently, the success of sports endeavors. Further research and the development of comprehensive support frameworks are essential to address the multifaceted nature of social support in the context of sports coaching.

## 5 Practical implications

The study presents important contributions on how sports coaches perceive different types of social support provided by various agents (i.e., family members, peers, and friends), as well as the consequences that social support will have on various aspects of the coach’s life (e.g., wellbeing). Future studies related to this topic could focus on: samples of sports coaches from different regions/countries, understanding how the cultural factor may affect the consequences of social support provided by different agents on coaches’ wellbeing; dividing the sample of coaches into male sports and female sports, following the trend of increasing female athletes in sports and creating knowledge associated with this evolution; dividing the sample of sports coaches by age groups, understanding how social support provided by different agents impacts the wellbeing of coaches of different ages. Thus, important conclusions can be drawn to help sports coaches make better use of social support, aiming to improve their general wellbeing and, consequently, their performance.

## Data Availability

The original contributions presented in the study are included in the article/supplementary material, further inquiries can be directed to the corresponding author/s.
